# Multi-institutional analysis of outcomes for thermo**s**phere **m**icrowave **a**blation treatment of **c**olorectal liver metastases: the **SMAC** study

**DOI:** 10.1007/s00330-021-08497-2

**Published:** 2022-01-29

**Authors:** Francesco De Cobelli, Marco Calandri, Angelo Della Corte, Roberta Sirovich, Carlo Gazzera, Paolo Della Vigna, Guido Bonomo, Gianluca Maria Varano, Daniele Maiettini, Giovanni Mauri, Nicola Camisassi, Stephanie Steidler, Francesca Ratti, Simone Gusmini, Monica Ronzoni, Luca Aldrighetti, Bruno C. Odisio, Patrizia Racca, Paolo Fonio, Andrea Veltri, Franco Orsi

**Affiliations:** 1 Department of Radiology, IRCCS San Raffaele Hospital, Milan, Italy; 2 Experimental Imaging Center, IRCCS San Raffaele Scientific Institute, Milan, Italy; 3 School of Medicine, Vita-Salute San Raffaele University, Milan, Italy; 4 Department of Oncology, University of Torino, Turin, Italy; 5 Interventional Radiology Unit, AOU San Luigi Gonzaga, Orbassano, Turin, Italy; 6 Department of Mathematics “Giuseppe Peano”, University of Torino, Turin, Italy; 7 Radiology Unit, AOU Città Della Salute E Della Scienza, Turin, Italy; 8 Divisione Di Radiologia Interventistica, Istituto Europeo Di Oncologia, Istituto Di Ricovero E Cura a Carattere Scientifico (IRCCS), Milan, Italy; 9 Dipartimento Di Oncologia Ed Emato-Oncologia, Università Degli Studi Di Milano, Milan, Italy; 10 Hepatobiliary Surgery Division, IRCCS San Raffaele Hospital, Milan, Italy; 11 Unit of Oncology, IRCCS San Raffaele Scientific Institute, Milan, Italy; 12 The University of Texas, Department of Interventional Radiology, MD Anderson Cancer Center, Houston, TX USA; 13 ColoRectal Cancer Unit, Department of Oncology, AOU Città Della Salute E Della Scienza, Turin, Italy; 14 Department of Surgical Sciences, University of Torino, Turin, Italy

**Keywords:** Colorectal neoplasms, Ablation, Microwaves

## Abstract

**Objectives:**

Oligometastatic colorectal cancer benefits of locoregional treatments but data concerning microwave ablation (MWA) are limited and interactions with systemic therapy are still debated. The aim of this study is to evaluate safety and effectiveness of Thermosphere™ MWA (T-MWA) of colorectal liver metastases (CLM) and factors affecting local tumor progression-free survival (LTPFS).

**Methods:**

In this multi-institutional retrospective study (January 2015–September 2019), patients who underwent T-MWA for CLM were enrolled. Complications according to SIR classification were collected, primary efficacy and LTP were calculated. Analyzed variables included CLM size at diagnosis and at ablation, CLM number, ablation margins, intra-segment progression, chemotherapy before ablation (CBA), variations in size (ΔS_DIA-ABL_), and velocity of size variation (V_DIA-ABL_) between CLM diagnosis and ablation. Uni/multivariate analyses were performed using mixed effects Cox model to account for the hierarchical structure of data, patient/lesions.

**Results:**

One hundred thirty-two patients with 213 CLM were evaluated. Complications were reported in 6/150 procedures (4%); no biliary complications occurred. Primary efficacy was achieved in 204/213 CLM (95.7%). LTP occurred in 58/204 CLM (28.4%). Six-, twelve-, and eighteen-month LTPFS were 88.2%, 75.8%, and 69.9%, respectively. At multivariate analysis, CLM size at ablation (*p* = 0.00045), CLM number (*p* = 0.046), ablation margin < 5 mm (*p* = 0.0035), and intra-segment progression (*p* < 0.0001) were statistically significant for LTPFS. ΔS_DIA-ABL_ (*p* = 0.63) and V_DIA-ABL_ (*p* = 0.38) did not affect LTPFS. Ablation margins in the chemo-naïve group were larger than those in the CBA group (*p* < 0.0001).

**Conclusion:**

T-MWA is a safe and effective technology with adequate LTPFS rates. Intra-segment progression is significantly linked to LTPFS. CBA does not affect LTPFS. Anticipating ablation before chemotherapy may take the advantages of adequate tumor size with correct ablation margin planning.

**Key Points:**

*• Thermosphere™-Microwave ablation is a safe and effective treatment for colorectal liver metastases with no registered biliary complications in more than 200 ablations.*

*• Metastases size at time of ablation, intra-segment progression, and minimal ablation margin < 5 mm were found statistically significant for local tumor progression-free survival.*

*• Chemotherapy before ablation modifies kinetics growth of the lesions but deteriorates ablation margins and does not significantly impact local tumor progression-free survival.*

**Supplementary Information:**

The online version contains supplementary material available at 10.1007/s00330-021-08497-2.

## Introduction

Colorectal cancer is the third most common tumor in both men and women. About a third of patients with a diagnosis of colorectal cancer during their lifetime will develop liver metastases (CLM) [1, 2] which represent a major prognostic determinant [3, 4].

In this field of treatment, specifically in the oligometastatic setting, locoregional treatments (LRTs) have recently been endorsed by NCCN [5] and ESMO guidelines [6] in the management of CLM. In the ESMO consensus guidelines, liver surgery, stereotactic body radiation therapy (SBRT), radiofrequency (RFA), microwave ablation (MWA), and trans-arterial therapies are all included in the LRTs toolbox, where physicians can select the best treatment for the right patient during tumor boards [6]. However, therapeutic indication varies according to the local expertise also due to the non-homogenous scientific evidence regarding the different LRTs. Furthermore, data concerning interaction of interventional treatments and systemic therapies are still limited [7, 8] or lacking.

Among image-guided ablation techniques, RFA is by far the most extensively investigated with milestone papers [9, 10]. MWA has been more recently proposed as an alternative ablation technique and is gaining popularity among interventional radiologists [11] as it represents some advantages over RFA, such as reduced “heat-sink” effect (lower heat dissipation in a vessel’s proximity) and larger ablation zones over a shorter period of time. The poor level of predictability of the ablation zone however is one of the well-known limitations of MWA probes. Thermosphere™ technology is a relatively recent advance of MWA whereby a combination of thermal control, field control, and wavelength control leads to the formation of a reliable spherical ablation zone [12]. This technique is currently investigated in the ongoing European prospective registry (CIEMAR-NCT03775980) and has already shown the potential of improving results of MWA in different clinical settings [13–15].

As recently highlighted [16], despite acknowledgments from the oncological guidelines and significant technical advancements, a relatively small number of studies have investigated the efficacy of MWA in the CLM setting and, specifically, no pertinent information is present about MWA with Thermosphere™ technology only (T-MWA). Furthermore, data are lacking about the interactions between ablative treatments and timing of concomitant systemic therapies and the growth kinetics of metastatic disease.

We therefore aimed at evaluating safety and effectiveness of T-MWA of CLM and factors affecting local tumor progression-free survival (LTPFS).

## Methods

### Study design

The study was approved by the IRBs of the three institutions (San Raffaele University Hospital, Milan (institution A); University of Torino (institution B); and Istituto Europeo di Oncologia, Milan (institution C)) in full respect of the Declaration of Helsinki and its later amendments. Informed consent was waived for this retrospective analysis.

Liver ablation registries of the three institutions were retrospectively evaluated and updated by review of the electronic medical records to select consecutive patients who underwent image-guided microwave ablation with Thermosphere™ technology for the treatment of CLM from January 2015 to September 2019.

### Ablation eligibility criteria, patient selection, and technique

Patient eligibility for ablation was discussed at each institutions’ multidisciplinary tumor boards. Patients underwent image-guided ablation if not amenable to surgery, in case of refusal to or in combination with surgery. In all three institutions, CLM were ablated if they did not exceed 5 cm in size and 5 lesions in number [17]. No oncological criteria were used for ablation eligibility.

Patients had to fulfill the following inclusion criteria: clinical and imaging evidence of CLM (radiological diagnosis of metastases on pre-operative dynamic contrast-enhanced imaging with a liver-specific acquisition protocol); availability of CT imaging at diagnosis and/or before ablation within 1 month from the procedure.

Percutaneous approach was free-hand ultrasound-guided or CT-guided (institutions A, B, and C) according to ultrasound conspicuity and local expertise, either under deep sedation (institutions A and B) or general anesthesia (institution C). Laparotomic and laparoscopic procedures (institutions A and C) were performed in the operating room with free-hand ultrasound guidance under general anesthesia.

All ablations were performed by experienced IRs in all centers using a 2450-MHz/100-W Microwave Emprint™ ablation system with Thermosphere™ technology (Medtronic).

Ablation protocol (power and time) was tailored to tumor size according to the manufacturer’s instructions (Ablation Zone Charts. R0065469, Instructions for Use, Emprint™).

### Collected data

For each patient, collected data were as follows: gender, age, TNM at diagnosis, mutational status (including KRAS and NRAS), location of primary CRC, clinical risk score [18], history of previous CLM resection or LRTs, chemotherapy before and after ablation, CLM number, and all the available imaging (pre- and post-procedure).

For each CLM, collected data included the following: size (at diagnosis and at ablation), intrahepatic location, time of appearance on imaging (synchronous/metachronous), amount of delivered energy over tumor size (*W* × *S*/mm).

CLM diameter at diagnosis and at the time of procedure was expressed both as a continuous variable and as a categorical entity (with cutoff values at 10, 15, 20, 25, 30, 35 mm). Available cross-sectional imaging from diagnosis to the time of ablation was reviewed in order to identify changes in size of the target lesions. Based on these data, information was acquired regarding degree of tumor size change from diagnosis to ablation (ΔS_DIA-ABL_) as well as velocity of size variation (size mm/time days, V_DIA-ABL_).

### Technique efficacy, tumor recurrence, and follow-up

Standardized terminology and reporting criteria for tumor ablation were used to determine ablation endpoints according to Ahmed et al. [19]. Complications were classified according to the SIR classification [20].

Technical success was assessed immediately after the procedure, by means of contrast-enhanced CT (for CT-guided procedures) or ultrasound evaluation (for US-guided procedures).

Technique efficacy was defined as absence of pathological enhancement at the ablation zone (residual tumor) on imaging (CT or MRI) at 1 month after ablation [21].

Minimal ablation margin evaluation on the three orthogonal planes was performed as previously described by Wang et al. [22] using the first cross-sectional contrast-enhanced imaging study following ablation.

The minimal ablation margin achieved in all three-dimensional axes was used to categorize the ablated CLM as having ≤ 5 mm or > 5 mm and ≤ 10 mm or > 10 mm minimal ablation margin.

Institutional follow-up for all three institutions included CT/MRI using a liver-specific acquisition protocol 1 month after the procedure, then every 3 months for the first year and every 6 months thereafter.

Local tumor progression (LTP) was defined as appearance of foci of vital disease at the edge of the ablation zone at any of the follow-up time points [19]. Intra-segment progression was defined as appearance of metastases within the same segment as the ablation area, at a minimum distance of > 1 cm from the ablated index lesion.

Intrahepatic progression was defined as appearance of metastases in any liver site outside of the ablation area. Data regarding overall survival (OS) were also recorded.

### Statistical analysis

Patient characteristics were described using standard descriptive statistics. Frequencies presented as percentages were used to express categorical values; median values with respective interquartile ranges were used for continuous variables.

Statistical analysis was performed with the open-source software R studio (R Foundation for Statistical Computing, www.r-project.org) by a qualified academic biostatistician (R.S.). Survival curves were generated with the Kaplan–Meier method and evaluated with the log-rank test. Univariate and multivariate analyses were performed using mixed effects Cox models, with random intercept per group in order to reduce to potential bias of multiple nodules per patient. Analysis was considered statistically significant if *p* < 0.05.

## Results

### Patient and CLM characteristics

**Fig. 1:**
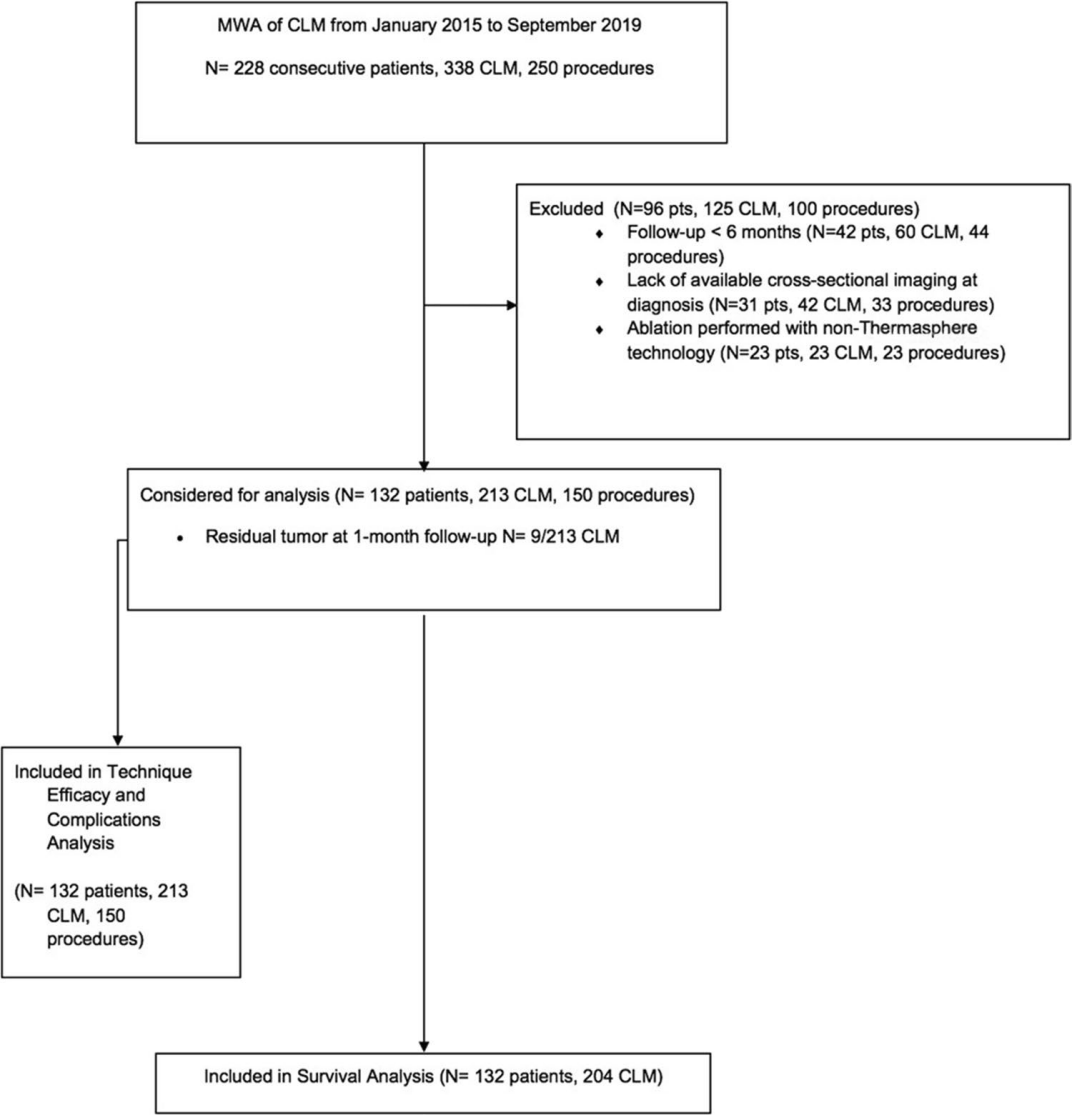
Flow diagram of patient selection and exclusion criteria. MWA, microwave ablation; CLM, colorectal liver metastases

During the study period, a total of 228 consecutive patients who underwent CLM ablation were identified. Patients with follow-up < 6 months (*n* = 42), lack of available cross-sectional imaging at diagnosis and/or the first CT control (*n* = 31), and CLM ablation performed with non-Thermosphere™ technology (*n* = 23) were excluded from analysis (Fig. 1).

After exclusions, a total of 132 patients (mean age 65.1 years [range 39–86]) who were deemed not to be surgical candidates (*n* = 82), refused surgery (*n* = 2), or underwent combined surgical/ablative approach (*n* = 48) were included in the analysis. Of these patients, 70 underwent chemotherapy after lesion identification prior to ablation (53%).

A total of 213 CLM were ablated in 150 procedures and included in the analysis (median size 1.4 cm [range 0.3–3.7]); 80 lesions were synchronous and 124 were metachronous.

The median follow-up period was 19 months (range 6–55 months). Patient and tumor characteristics at baseline are summarized in Table 1.

**Table 1:** Baseline characteristics. *EHD*, extrahepatic disease; *CT*, computed tomography; *CEA*, carcinoembryonic antigen; *CRC*, colorectal cancer; *CLM*, colorectal liver metastases; *LRT*, locoregional treatment; *TARE*, trans-arterial radioembolization; *RFA*, radiofrequency ablation; *RECIST*, Response Evaluation Criteria in Solid Tumors; *PD*, progressive disease; *SD*, stable disease; *PR*, partial response

Patients (*N* = 132)						
Gender		AllRas mutation	43 (32.6%)		Location of primary tumor	
Male	86 (65.2%)	AllRas wt	61 (46.2%)		Cecum	8 (6.1%)
Female	46 (34.8%)	Missing	28 (21.2%)		Ascending colon	19 (14.4%)
Age (years) > 70	45 (34.1%)	KRAS mut	42 (31.8%)		Transverse colon	3 (2.3%)
> 5 Lesions	26 (19.7%)	KRAS wt	68 (51.5%)		Descending colon	26 (19.7%)
EHD at time of ablation	34 (25.8%)	Missing	22 (16.67%)		Sigmoid colon	35 (26.5%)
Indication for ablation		NRAS mut	4 (3%)		Rectum	40 (30.3%)
Non-surgical candidate	82 (61.1%)	NRAS wt	98 (74.2%)		Missing	1 (0.8%)
Surgery refusal	2 (1.5%)	Missing	30 (22.7%)		Nodal status of primary	
Combined approach	48 (36.4%)	CEA at diagnosis of primary tumor	81.8 ± 933.7 (1.3–7288.6)		0	35 (26.5%)
Technical approach		CEA at time of ablation	7.7 ± 24.2 (0.8–118.5)		1	59 (44.7%)
Percutaneous	76 (57.6%)	Clinical risk score			2	33 (25%)
Laparoscopic	17 (12.9%)	0	6 (4.5%)		Missing	5 (37.9%)
Laparotomic	38 (28.8%)	1	14 (10.6%)		Grade of primary tumor	
Imaging guidance		2	19 (14.4%)		G1	4 (3%)
Ultrasound	95 (72.5%)	3	32 (24.2%)		G2	82 (31.8%)
CT	36 (27.3%)	4	6 (4.5%)		G3	23 (17.4%)
Lesions ablated per session		5	1 (0.8%)		Missing	23 (17.4%)
Single	88 (66.7%)	Missing	54 (40.9%)		Previous hepatic resection	38 (28.8%)
Multiple	44 (33.3%)				Post-ablation chemotherapy	61 (46.2%)
CLM (*N* = 204)						
Size at ablation (mm)^†^	15.4 ± 8 (3–37)	Subcapsular location		54 (25.4%)	Synchronous	83 (39%)
Size at CSI	14.8 ± 8.2 (3–46)	Proximity to vessels > 3 mm		79 (37.1%)	Metachronous	130 (61%)
Largest lesion size at any time	18.4 ± 9.5 (3–47)	Chemotherapy after lesion discovery and before ablation		112 (52.6%)	Time from lesion discovery to ablation (days)	160.6 ± 158.8 (0–779)
Previous LRT	41 (19.2%)	Time from last hepatic resection to ablation		406.7 ± 382.5 (5–1464)	Response to pre-ablation chemotherapy (RECIST1.1)	
TARE	3 (1.4%)	Time from last chemotherapy cycle to ablation (days)		68.17 ± 60.55 (0–381)	PD	28 (29.5%)
RFA	28 (13.1%)				SD	11 (11.6%)
Resection	10 (4.7%)				PR	56 (58.9%)

Comparison between institutions A, B, and C revealed homogeneity in most of the analyzed data such as gender, presence of extrahepatic disease at time of ablation, number of ablations in a single session, post-ablation chemotherapy, KRAS and NRAS mutations, localization and nodal status of primary tumor, CEA level at time of ablation, subcapsular localization, time from lesion discovery to ablation, chemotherapy after lesion discovery and before ablation, incomplete ablation, and LTP rates. The evaluation regarding similarities and differences among institutions is available as supplementary material (Tables S1 and S2).

### Local, hepatic, and patient outcomes

Primary efficacy was observed in 204/213 nodules (95.7%). Of the 9 cases with incomplete ablation, one underwent repeated ablation and 4 refused further local treatments and underwent chemotherapy; one case had a multifocal relapse observed at 1-month follow-up and was switched to chemotherapy; in the remaining 3 cases, surgical resection was performed on the incompletely ablated nodule.

No fatal events occurred after ablation. Complications were observed in 6/150 procedures (4%), of which three were classified as minor (two non-bleeding hematomas which did not require further treatment and one case with a collection which required antibiotic therapy and 3 were classified as major (three cases of pleural effusion managed with drainage placement).

LTP was observed in 58/204 CLM during the follow-up period (28.4%). Six-, twelve-, and eighteen-month LTPFS were 88.2%, 75.8%, and 69.9%, respectively. Twelve out of 58 LTPs underwent re-ablation (20.7%), whereas 31 (53.4%) were scheduled to receive chemotherapy due to multifocal liver progression, 5 (8.6%) underwent surgical resection, and 10 (17.2%) refused further treatments.

Intrahepatic progression was observed in 79/132 patients during the follow-up period (59.8%). Six, twelve, and eighteen months, intrahepatic progression-free survival rates were 65.8%, 56%, and 36.8%, respectively.

Cancer-related deaths occurred in 14/132 patients during the follow-up period (10.6%). Twelve- and twenty-four-month OS were 98.3% and 89.9%, respectively.

### Univariate and multivariate analyses

At univariate analysis (Table 2), risk factors for occurrence of LTP were CLM size at ablation (HR = 1.08, *p* = 0.0001), CLM size at ablation > 2 cm (HR = 2.55, *p* = 0.00157), CLM size at CSI diagnosis (HR = 1.06, *p* = 0.0008), largest CLM size at any time point before ablation (HR = 1.07, *p* < 0.0001), and intra-segment progression (HR = 3.05, *p* < 0.0001). Margins ≥ 5 mm (HR = 0.28, *p* = 0.0025) and margins > 10 mm (HR = 0.24, *p* = 0.0014) were both protective factors for LTP.

**Table 2:** LTPFS mixed effects Cox model. *LTPFS*, local tumor progression-free survival; *HR*, hazard ratio; *CEA*, carcinoembryonic antigen; *CLM*, colorectal liver metastases; *LRT*, locoregional therapy; *RECIST*, Response Evaluation Criteria in Solid Tumors; *PD*, progressive disease; *SD*, stable disease; *PR*, partial response

Variable	Univariate				Multivariate			
	Coefficient	HR	Standard error	*p*	Coefficient	HR	Standard error	*p*
Gender	0.48	1.62	0.37	0.2				
Age > 70	0.23	1.26	0.35	0.52				
Center (baseline B)								
A	− 0.41	0.66	0.49	0.41				
C	0.09	1.1	0.47	0.85				
Extrahepatic disease at time of ablation	− 0.42	0.65	0.43	0.33				
Percutaneous (baseline)								
Laparoscopic	− 0.5	0.60	0.49	0.3				
Open	− 0.75	0.47	0.41	0.07				
Imaging guidance	− 0.44	0.65	0.36	0.23				
Number of lesions ablated per session	0.03	1.03	0.15	0.84	0.06	1.07	0.03	0.046
> 1 lesion ablated	0.48	1.62	0.34	0.16				
> 3 lesions ablated	0.89	2.44	0.47	0.056				
Hepatic resection (baseline none)								
Pre-ablation	0.63	1.88	0.39	0.11				
Post-ablation	− 0.09	0.91	0.55	0.87				
Time from hepatic resection to ablation	0.0014	1.001	0.0012	0.23				
Post-ablation chemotherapy	− 0.39	0.67	0.35	0.26				
AllRas mutation	− 0.34	0.71	0.41	0.4				
KRAS	− 0.15	0.86	0.42	0.73				
NRAS	− 21.26	0	3.4768	1				
CEA at diagnosis of primary tumor	− 0.00023	1	0.00047	0.63				
CEA at diagnosis of primary tumor > 200 ng/mL	0.06	1.06	0.78	0.94				
CEA at time of ablation	− 0.009	0.99	0.012	0.45				
Clinical risk score								
1	− 0.69	0.5	0.96	0.47				
2	− 0.72	0.49	0.8	0.4				
3	− 0.89	0.41	0.82	0.27				
4	− 0.08	0.92	0.92	0.93				
5	− 18.8	0	15.988	1				
Location of primary tumor (baseline cecum)								
Ascending	0.75	2.12	0.76	0.32				
Transverse	− 20.02	0	24.134	1				
Descending	0.34	1.4	0.72	0.64				
Sigma	0.2	1.22	0.71	0.78				
Rectum	− 0.25	0.77	0.72	0.72				
Nodal status of primary tumor								
1	− 0.14	0.87	0.5	0.78				
1a	− 1.34	0.26	0.8	0.093				
1b	− 0.04	0.96	0.54	0.94				
1c	0.99	2.69	0.7	0.16				
2	0.35	1.42	0.65	0.59				
2a	− 0.13	0.88	0.55	0.81				
2b	− 0.12	0.89	0.8	0.89				
Metastatic disease at diagnosis	− 0.15	0.86	0.36	0.67				
CLM size at ablation	0.08	1.08	0.021	**0.0001**	0.1	1.1	0.02	0.00045
CLM size at diagnosis	0.056	1.06	0.017	**0.0008**				
Largest CLM size	0.07	1.07	0.016	**0.00006**				
Amount of delivered energy	0.00009	1	0.0001	0.43				
Synchronous	− 0.15	0.86	0.36	0.67				
Subcapsular location	0.21	1.23	0.36	0.57				
Proximity to vessels > 3 mm	0.3	1.35	0.32	0.34				
Any LRT before ablation	0.7	2.01	0.42	0.096				
Time from lesion discovery to ablation (days)	0.0015	1.001	0.001	0.15				
Chemotherapy after lesion discovery and before ablation	0.42	1.52	0.34	0.22				
Time from last chemotherapy cycle to ablation	− 0.007	0.99	0.005	0.17				
Response to pre-ablation chemotherapy (RECIST1.1) (baseline PD)								
SD	0.79	2.21	0.82	0.33				
PR	− 0.12	0.89	0.5	0.81				
Margin (baseline < 5 mm)								
≥ 5 mm	− 1.26	0.28	0.42	**0.0025**	− 1.03	0.36	0.35	0.0035
> 10 mm	− 1.41	0.24	0.44	**0.0014**				
Intrasegment progression	1.11	3.05	0.29	0.00009	1.4	4.1	0.36	0.00009

Bold values are statistically significant (*p* < 0.05)

At the multivariate analysis (Table 2), a balanced model for LTPFS including CLM number (*p* = 0.046), size at ablation (*p* = 0.00045), minimal ablation margin ≥ 5 mm (*p* = 0.0035), and intra-segment progression (*p* < 0.0001) was created.

### Lesion size and growth kinetics analysis

CLM size at diagnosis on cross-sectional imaging was 14.8 ± 8 mm (3–46 mm); CLM size at time of ablation was 15.4 ± 8 mm (3–37 mm). Mean time from detection to ablation was 160.6 ± 158.8 days (0–779).

CLM size both at time of ablation and at time of diagnosis was a statistically significant predictor of LTP, analyzed as a continuous variable as well as using different thresholds (HR varying from 1.08 to 4.74, *p* varying from 0.0001 to 0.022, see Table 3).

**Table 3:** LTPFS univariate mixed effects Cox model on size. *LTPFS*, local tumor progression-free survival; *HR*, hazard ratio; *ΔS*_*DIA-ABL*_, size change from diagnosis to ablation; *V*_*DIA-ABL*_, velocity of size change from diagnosis to ablation

Variable	Coefficient	HR	Standard error	*p*
Size at ablation	0.08	1.08	0.02	**0.0001**
Size at ablation > 10 mm	1.36	3.88	0.39	**0.0005**
Size at ablation > 15 mm	0.99	2.68	0.33	**0.003**
Size at ablation > 20 mm	1.06	2.89	0.34	**0.002**
Size at ablation > 25 mm	1.29	3.65	0.4	**0.0014**
Size at ablation > 30 mm	0.75	2.12	1.1	0.5
Size at ablation > 35 mm	− 19.11	0	23.030	1
Size at diagnosis	0.06	1.06	0.02	**0.0008**
Size at diagnosis > 10 mm	1.52	4.58	0.4	**0.00016**
Size at diagnosis > 15 mm	0.95	2.58	0.3	**0.0034**
Size at diagnosis > 20 mm	0.5	1.65	0.4	0.22
Size at diagnosis > 25 mm	0.83	2.29	0.46	0.07
Size at diagnosis > 30 mm	1.19	3.29	0.52	**0.022**
Size at diagnosis > 35 mm	1.56	4.74	0.65	**0.017**
ΔS_DIA-ABL_	0.06	1.01	0.02	0.78
ΔS_DIA-ABL_ > 0 mm	− 0.043	0.96	0.32	0.89
V_DIA-ABL_	− 1.84	0.16	1.8	0.3
V_DIA-ABL_ > 0 mm/day	− 0.21	0.8	0.3	0.53

Bold values are statistically significant (*p* < 0.05)

Mean ΔS_DIA-ABL_ was 0.8 ± 8.8 mm (− 31/ + 22); mean V_DIA-ABL_ was 0.06 ± 0.2 mm/days (− 0.16/ + 2.29). No association was seen between LTPFS and ΔS_DIA-ABL_ (*p* = 0.78) or V_DIA-ABL_ (*p* = 0.3).

Chemotherapy before ablation (CBA) was administered in one hundred twelve out of 204 CLM (70 patients). Regimens administered included the following: *n* = 5 capecitabine; *n* = 2 capecitabine + bevacizumab; *n* = 2 folfiri, *n* = 11 folfiri + bevacizumab, *n* = 4 folfiri + cetuximab, *n* = 9 folfox, *n* = 14 folfox + bevacizumab, *n* = 10 xelox; *n* = 9 folfox + panitumumab, *n* = 4 folfoxiri + bevacizumab. Time elapsed between detection and ablation was longer in CLM undergoing CBA (240 ± 170.8 days vs 63 ± 62 days, *p* < 0.001). Differences and similarities between the CBA and chemo-naïve groups are summarized in Table 4.

**Table 4:** CLM characteristics according to subgroups based on pre-ablation chemotherapy. *CLM*, colorectal liver metastases; *CBA*, chemotherapy before ablation; *CSI*, cross-sectional imaging

Variables	CBA (*N* = 112)	Chemo-naive (*N* = 92)	*p*
Size at ablation (mm)	13.4 ± 7 (3–35)	17.5 ± 8 (5–37)	** < 0.0001**
Size at CSI	16.8 ± 9 (3–46)	13 ± 6 (4–30)	**0.015**
Amount of delivered energy (*W* × s/mm)	2253.56 ± 1340.25 (545–10,000)	2179.5 ± 1148 (700–7125)	0.6
Subcapsular location	24 (21.4%)	28 (30.4%)	0.15
Proximity to vessels > 3 mm	41 (36.6%)	34 (37%)	0.53
Margin < 5 mm	43 (38.4%)	23 (25%)	**0.035**
Margin 5–10 mm	46 (41%)	27 (29.35%)	0.82
Margin > 10 mm	23 (20.6%)	42 (45.65%)	** < 0.0001**
Local tumor progression	36 (32.14%)	22 (23.9%)	0.2

Bold values are statistically significant (*p* < 0.05)

LTP was observed in 36/112 CLM undergoing CBA (32.1%) and 22/92 chemo-naïve CLM (23.9%, *p* = 0.21). At univariate analysis, CBA was not associated with LTPFS (*p* = 0.22).

**Fig. 2:**
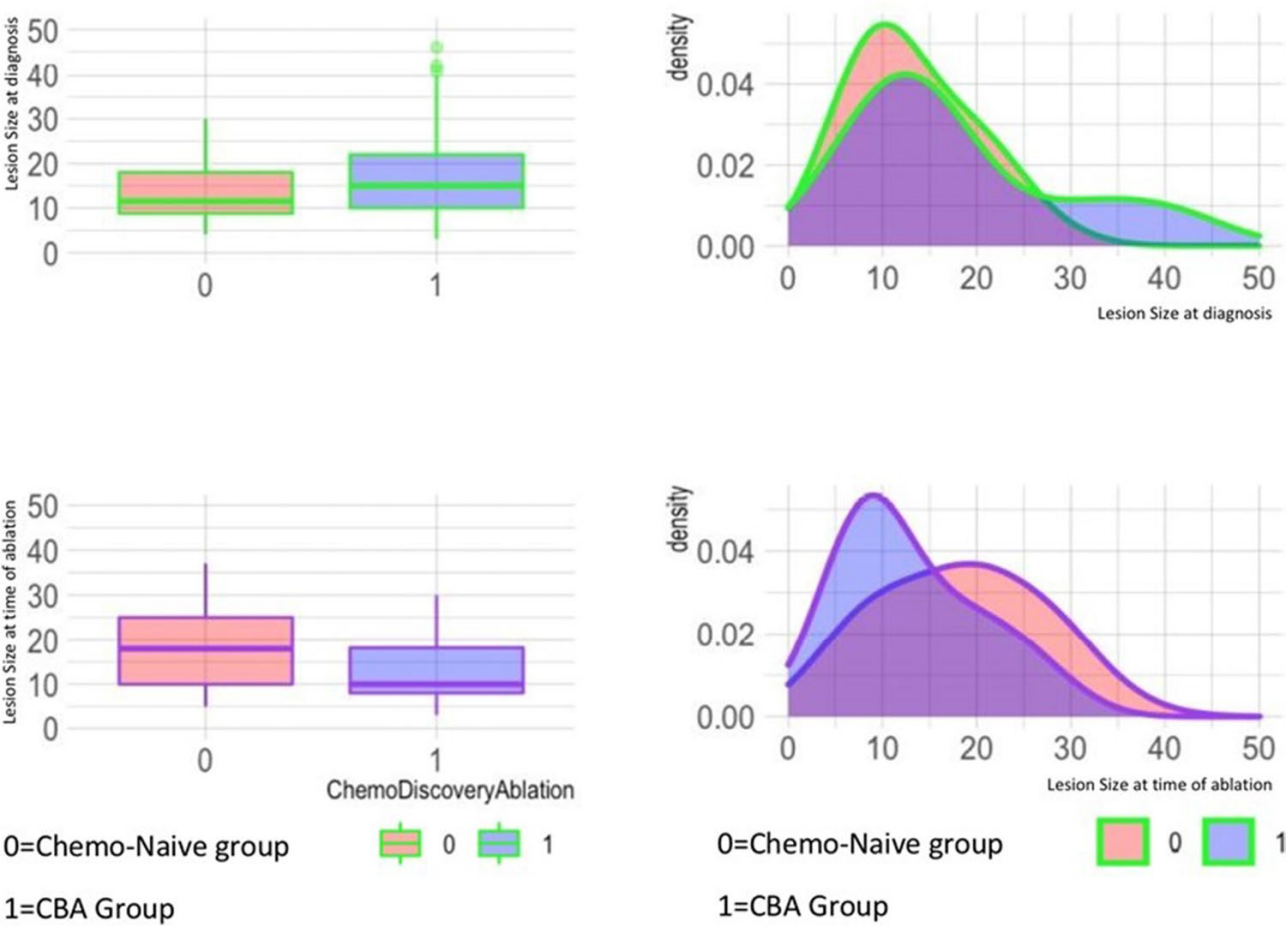
Lesion size at time of diagnosis and at time of ablation. The population was studied according to chemo-treated lesions or chemo-naïve lesions. CBA, chemotherapy before ablation

At time of diagnosis, lesion size was slightly higher in CLM receiving CBA (16.8 ± 9 mm vs 13 ± 6 mm, *p* = 0.015), whereas at time of ablation, size was higher in chemo-naive CLM (17.5 ± 8 vs 13.4 ± 7, *p* < 0.001). No statistical difference was observed between size at diagnosis in the CBA group and size at ablation in chemo-naive CLM (chemo-treated 16.8 ± 9 mm; chemo-naive 17.5 ± 8, *p* = 0.45). Size distribution at diagnosis and at ablation according to subgroups based on history of CBA is reported in Fig. 2. Margins in the chemo-naïve groups were significantly larger than those in the CBA group (margins < 5 mm groups, *p* = 0.035; margins > 10 mm groups, *p* < 0.0001).

**Fig. 3:**
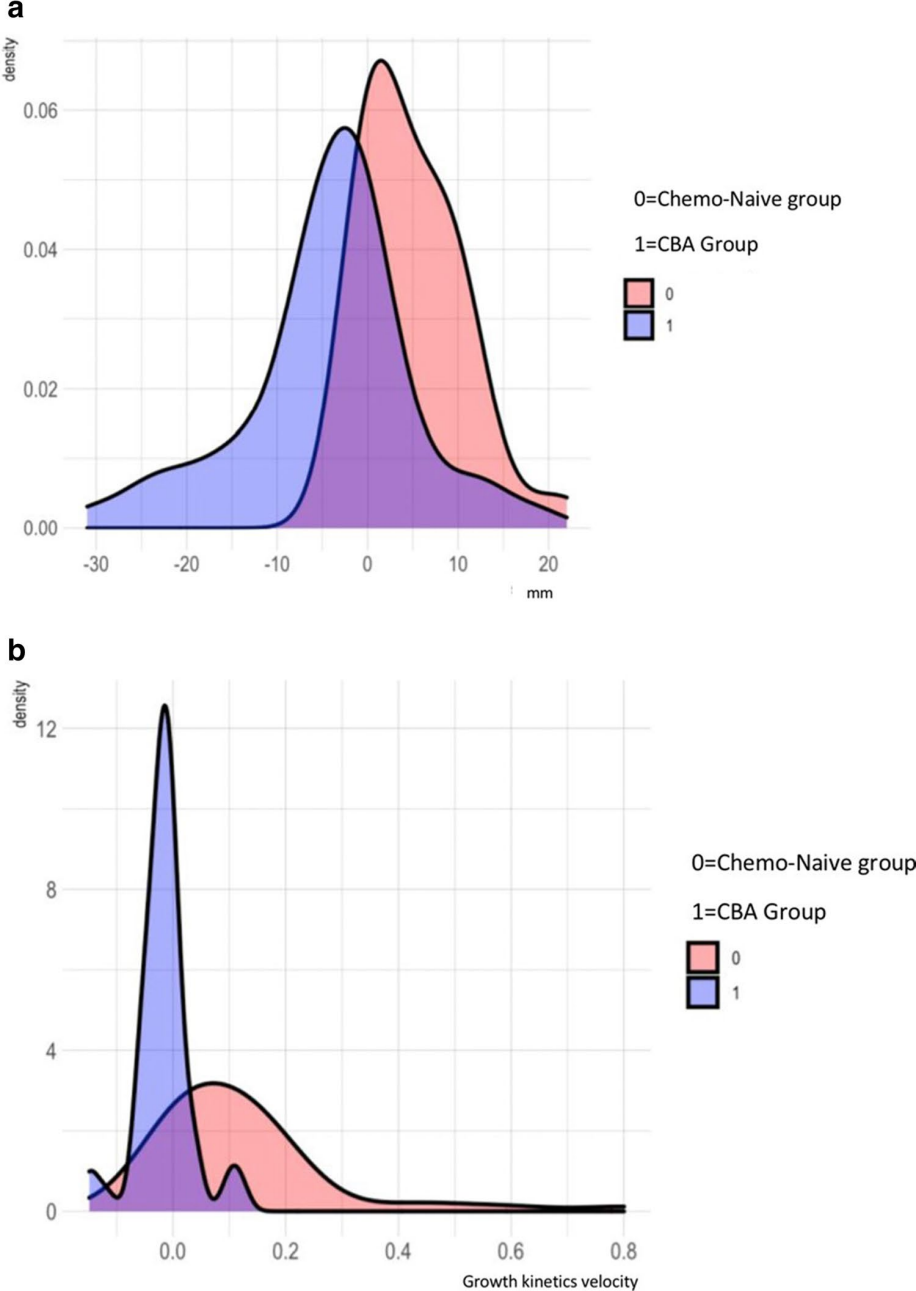
**a** Distribution of ΔS_DIA-ABL_ according to chemo-treated or chemo-naïve lesions; chemotherapy slightly reduces the lesion size of the lesion (4.8 ± 5.4 mm vs − 2.9 ± 9.7 mm, *p* < 0.001); however, this did not affect LTP outcomes. CBA, chemotherapy before ablation. **b** Distribution of V_DIA-ABL_ according to chemo-treated or chemo-naïve lesions; chemotherapy slightly reduce the kinetics growth of the lesion (0.13 ± 0.28 vs − 0.007 ± 0.06, *p* < 0.0001). CBA, chemotherapy before ablation

**Fig. 4:**
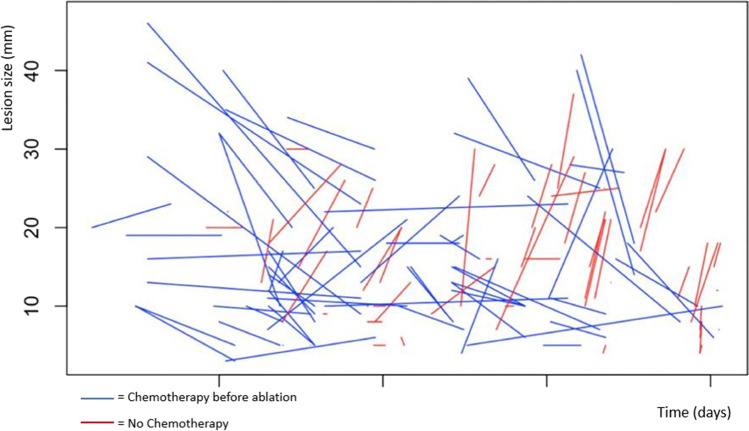
Growth kinetics of lesions before ablation: red lines represent chemo-naïve lesions; blue lines chemo-treated lesions. For each line, point A represents lesion size at diagnosis whereas point B represents lesion size at ablation. As a result, the slope of each line is a graphical representation of velocity of growth. Almost all the chemo-naïve lesions are fastly growing, whereas chemo-treated lesions had a more mixed response, most of them remained stable, some reduced in size, a few of them increased in size. V_DIA-ABL_ and ΔS_DIA-ABL_ were statistical significant among groups; however, they did not impact on LTPFS (ΔS_DIA-AB_L *p* = 0.63, V_DIA-ABL_ *p* = 0.38)

ΔS_DIA-ABL_ and V_DIA-ABL_ were significantly higher in CLM not undergoing CBA (ΔS_DIA-ABL_: 4.8 ± 5.4 mm vs − 2.9 ± 9.7 mm, *p* < 0.001; V_DIA-ABL_: 0.13 ± 0.28 vs − 0.007 ± 0.06, *p* < 0.001). Distribution of ΔS_DIA-ABL_ and V_DIA-ABL_ is shown in Fig. 3 and depicted for each single lesion in Fig. 4.

## Discussion

Our multicenter study gives pertinent information regarding T-MWA and analyzes the lesion size in a dynamic approach, investigating the kinetics growth before ablation and factors that can modify it.

Briefly, our findings highlight that:

- T-MWA is a safe technique (2% of major complications, with no biliary complications) with satisfying LTPFS rates at 12 months (75.8%);
- Multivariate analysis with Cox regression confirms lesion size, minimum margins size, and number of hepatic lesions < 3 as factors influencing outcome and unveils a relationship between intra-segment progression and LTPFS;
- Lesion size clearly impacts outcome, also when measured at time of diagnosis (*p* = 0.000237);
- Chemotherapy before ablation modifies growth kinetics (both ΔS_DIA-ABL_ and V_DIA-ABL_) but deteriorates ablation margin width and does not significantly impact LTPFS (*p* = 0.37).

Literature concerning microwave ablation of colorectal liver metastases or factors impacting outcome is relatively few if compared with other therapies present in the ESMO toolbox (in primis, RFA, or surgery). T-MWA, introduced in the clinical practice in 2015, has gained acceptance as a valid tool for treatment and is recently being investigated in the first European prospective registry (CIEMAR) whose results are expected in 2025. Moreover, a phase III single-blind prospective randomized controlled trial (the COLLISION trial [23]) is ongoing with the objective to prove non-inferiority of MWA compared to hepatic resection in patients with at least one resectable and ablatable CLM and no extrahepatic disease. Thus, our paper represents the first multicenter retrospective analysis of results achieved in the clinical practice with the application of T-MWA in the treatment of CLM.

According to our results, T-MWA is a safe technology with LTPFS rates of 88.2%, 75.8%, and 69.9% at 6, 12, and 18 months. These results are comparable with recently published data in a historic single center RFA cohort [9] and comparable with other previously published cohorts [24].

Concerning adverse events, major complications (listed according to SIR classification) occurred in 2% of the procedures, with no related deaths. This is consistent (even inferior) with other MWA series, such as the 2.6% according to Liang et al. [25]. Furthermore, although the site of the lesions was not classified as peri-hilar or not in our study, no biliary complications were accounted for after T-MWA procedures; this is a relevant finding, especially if compared with other literature reports concerning MWA technologies with higher numbers of reported biliary ducts injuries [26].

Multivariate analysis confirms lesion size, minimum margin size, and the number of hepatic lesions as factors affecting LTPFS. This is consistent with the published data concerning the RFA procedures [22, 24, 27–29], once more highlighting the prominent role of accurate patient selection and the need of an effective interventional technique.

To the best of our knowledge, for the first time, intra-segment progression was evaluated in the analysis and found statistically significant in a multivariate model (while hepatic progression outside the ablated lesion was not). Intra-segment progression was defined as the appearance of tumor foci in the segment of the ablated lesion apart from the edges of the ablation scar with a minimum distance of 1 cm. Although intra-segment progression cannot be used as a predictor (obviously it was observed during follow-up), the relationship with LTP focuses the attention at the local level rather than at a systemic level, highlighting the possibility of a correlation between LTP and a locally aggressive behavior of the tumor. Although we could not demonstrate in this series an independent role of RAS mutational status as demonstrated in other series [30–32], this finding concerning the intra-segment recurrence is in line with surgical literature and histopathological research that identifies and describes different local histopathological growth patterns [33] even on molecular basis [34]. Overall, from the interventional perspective, these data stress the importance of adequate minimum margins of ablations, at least of 5 mm according to our data (and consistently with the literature [22]).

Lesion size was found as an independent predictor both at time of diagnosis and at time of ablation. Furthermore, ΔS_DIA-ABL_ and V_DIA-ABL_ were not found to be predictors of different LTPFS. Although V_DIA-ABL_ tended to be higher in the group without chemotherapy, CBA was not found as an independent predictor for LTPFS; chemotherapy did slightly reduce the kinetic growth of the lesion. However, mean lesion sizes at ablation and at time of diagnosis (in both subgroups) were under 2 cm and therefore clearly amenable to ablative treatments. We did not find any statistical difference between size in chemo-treated CLM at diagnosis and size in chemo-naive CLM at ablation.

Even though statistical significance was not achieved, higher LTP rates were observed in the CBA group compared to the chemo-naïve group (32.1 vs 23.9%). Furthermore, ablation margins were statistically worse in the CBA group compared to the chemo-naïve group despite similar amount of delivered energy and ablation sites. This finding can be related with the changes in liver parenchyma texture and enhancement during chemotherapy, with subsequent difficulties in targeting the index lesions during the procedure [35–37].

To date, therapeutic decision-making regarding the use of thermal ablation is mostly left to multidisciplinary tumor board consultations and local expertise. Our data provide new insights on the interaction between chemotherapy and T-MWA [6]. According to the phase II CLOCC trial [38] as well as a metanalysis by Meijerink et al. [39], there is a synergistic effect between systemic and local therapies that improve long-term overall survival; in the CLOCC trial, thermal ablation was performed upfront in the RFA plus chemotherapy group and only 6 patients had previously received a chemotherapeutic regimen.

Few and inconclusive data [40] exist in literature concerning the oncological management of CLM prior to local treatment and in particular prior to ablation. Benhaim et al. [8] demonstrated that RFA, for CLM initially greater than 25 mm, but downsized by neo-adjuvant chemotherapy, is associated with increased rate of LTP. Indeed, the persistence of microscopic residual disease, not visible at the radiological evaluation, following CBA may lead to some difficulties in the correct planning of ablative margins (a concept similar to that of missing metastases [41]). Accordingly, our data did not find a significant role of CBA on LTPFS with LTP rates slightly superior in those who received CBA; furthermore, adequate ablation margins were more frequently observed in the chemo-naïve group. Lastly, lesion size was an independent factor already at time of diagnosis.

Taken together, our data underline the potential role of treating CLM in a short timeframe following diagnosis and suggest thermal ablation upfront with respect to chemotherapy. Although many questions remain open and evidence should be more robust, in consideration of these findings and those already present in the literature [8, 38–40], we believe that ablation upfront to chemotherapy should be a concept to be highly considered by interventional radiologists and carefully evaluated during tumor board evaluations, by introducing the ASARA principle: to deliver ablative treatments for CLM **A**s **S**oon **A**s **R**easonably **A**chievable.

Our paper has some limitations. First of all, the follow-up time is relatively short if compared with other papers [9, 24, 27] on CLM ablation. However, the Thermosphere™ technology was launched at the end of 2014, and in general, MWA is a younger technology compared to RFA. Although 19 months of mean follow-up may not appear very long, the CIEMAR registry (endorsed by CIRSE, on the same technology and disease) has its primary endpoint (local tumor control) at 12 months after treatment.

In patients receiving chemotherapy prior to ablation, regimens were those indicated by ESMO/NCCN guidelines [5, 6], in particular according to the molecular profiles of the diseases (doublet or triplet regimens plus bevacizumab or anti-EGFR agents). This does not make this treatment subgroup uniform but it correctly reflects current practice and patient management.

Differently from other series, we did not find any statistical significance by Ras mutational status. This result may be affected by data incompleteness related to the retrospective nature of this study.

Lastly, in our study cohort, lesion site was not classified as peri-hilar or not, and this may generate a bias when assessing bilary complications.

In conclusion, T-MWA seems an effective and safe technology for CLM treatment, with no biliary complications in more than 200 ablated lesions. LTPFS is linked to intra-segment recurrence, highlighting the potential relationship with the tumor biology and its local aggressiveness. Care should be exercised in choosing the correct timing of the ablation: anticipating ablation As Soon As Reasonably Achievable (ASARA) may allow proper planning of ablation margins without losing the potential benefits of post-ablation chemotherapy.

## Supplementary Information

Below is the link to the electronic supplementary material.

Supplementary file1 (DOCX 30 KB)

## Abbreviations

CBA: Chemotherapy before ablation
CLM: Colorectal liver metastases
CT: Computed tomography
LRT: Locoregional therapies
LTP: Local tumor progression
LTPFS: Local tumor progression-free survival
MRI: Magnetic resonance imaging
MWA: Microwave ablation
OS: Overall survival
RFA: Radiofrequency ablation
SBRT: Stereotactic body radiation therapy
T-MWA: Thermosphere microwave ablation
V_DIA-ABL_: Velocity of size change from diagnosis to ablation
ΔS_DIA__-ABL_: Degree of size change from diagnosis to ablation

## Declarations

### Guarantor

The scientific guarantor of this publication is Marco Calandri.

### Statistics and biometry

One of the authors (R.S.) has significant statistical expertise.

### Methodology

• Retrospective

• Observational

• Multicentre study
