# Supplementary material for: Multi-institutional analysis of outcomes for thermosphere microwave ablation treatment of colorectal liver metastases: the SMAC study
Source: Eur Radiol. 2022 Jan 29;32(6):4147–59. doi: 10.1007/s00330-021-08497-2 (PMC9123066; doi:10.1007/s00330-021-08497-2)
Supplement: Supplementary file 1 — Supplementary file1 (DOCX 30 KB) [file 330_2021_8497_MOESM1_ESM.docx]

| **Table S1 - Patient Characteristics** | | | |  |  |
| --- | --- | --- | --- | --- | --- |
| **Variables** | **INSTITUTION A (N=48)** | **INSTITUTION B (N=33)** | **INSTITUTION C (N=51)** | **TOTAL (N=132)** | **P** |
| GENDER |  |  |  |  |  |
| Male | 26 (54.2%) | 24 (72.7%) | 36 (70.6%) | 86 (65.2%) | 0.13 |
| Female | 22 (45.8%) | 9 (27.3%) | 15 (29.4%) | 46 (34.8%) |  |
| Age (years) >70 | 13 (27.1%) | 20 (60.6%) | 12 (23.5%) | 45 (34.1%) | <0.001 |
| Extrahepatic diseases at time of ablation | 10 (20.8%) | 7 (21.2%) | 17 (33.3%) | 34 (25.8%) | 0.3 |
| INDICATION FOR ABLATION |  |  |  |  |  |
| Non-Surgical Candidate | 16 (33.3%) | 33 (100%) | 33 64.7%) | 82 (61.1%) | <0.001 |
| Surgery Refusal | 1 (2.1%) | 0 | 1 (2%) | 2 (1.5%) |  |
| Combined Approach | 31 (64.6%) | 0 (0%) | 17 (33.3%) | 48 (36.4%) | <0.001 |
| TECHNICAL APPROACH |  |  |  |  |  |
| Percutaneous | 12 (25%) | 33 (100%) | 32 (62.7%) | 76 (57.6%) | <0.001 |
| Laparoscopic | 15 (31.3%) | 0 (0%) | 2 (3.9%) | 17 (12.9%) | <0.001 |
| Laparotomic | 21 (43.7%) | 0 (0%) | 17 (33.3%) | 38 (28.8%) | <0.001 |
| IMAGING GUIDANCE |  |  |  |  |  |
| Ultrasound | 47 (97.9%) | 31 (93.8%) | 18 (35.3%) | 95 (72.5%) | <0.001 |
| CT | 1 (2.1%) | 2 (6.1%) | 33 (64.7%) | 36 (27.3%) | <0.001 |
| LESIONS ABLATED PER SESSION |  |  |  |  |  |
| Single | 32 (66.7%) | 23 (69.7%) | 33 (64.7%) | 88 (66.7%) | 0.9 |
| Multiple | 16 (33.3%) | 10 (30.3%) | 18 (35.3%) | 44 (33.3%) |  |
| Previous Hepatic Resection | 9 (18.8%) | 6 (18.2%) | 23 (45.1%) | 38 (28.8%) | 0.006 |
| Post-ablation Chemotherapy | 23 (47.9%) | 19 (57.6%) | 19 (37.3%) | 61 (46.2%) | 0.19 |
| AllRas mutation | 20 (41.7%) | 11 (33.3%) | 15 (23.5%) | 43 (32.6%) | 0.43 |
| KRAS | 18 (37.5%) | 9 (27.3%) | 15 (29.4%) | 42 (31.8%) | 0.6 |
| NRAS | 2 (4.2%) | 2 (6.1%) | 0 | 4 (3%) | 0.18 |
| CEA at time of ablation | 5.3±27.7 (1-83.6) | 9±20.7 (0.8-114) | 9.1±26 (1.2-118.5) | 7.7±24.2 (0.8 – 118.5) | 0.7 |
| Clinical Risk score |  |  |  |  |  |
| 0 | 1 (2.1%) | 4 (12.1%) | 1 (2%) | 6 (4.5%) | 0.56 |
| 1 | 2 (4.2%) | 10 (30.3%) | 2 (4%) | 14 (10.6%) | 0.04 |
| 2 | 5 (10.4%) | 9 (27.3%) | 5 (9.8%) | 19 (14.4%) | 0.71 |
| 3 | 17 (35.4%) | 8 (24.2%) | 7 (13.7%) | 32 (24.2%) | 0.008 |
| 4 | 1 (2.1%) | 0 | 5 (9.8%) | 6 (4.5%) | 0.003 |
| 5 | 0 | 0 | 1 (2%) | 1 (0.8%) | 0.27 |
| Missing | 22 (45.8%) | 2 (6.1%) | 30 (58.8%) | 54 (40.9%) | <0.001 |
| GRADE OF PRIMARY TUMOR |  |  |  |  |  |
| G1 | 2 (4.2%) | 0 | 2 (4%) | 4 (3%) | 0.83 |
| G2 | 41 (85.4%) | 14 (42.4%) | 27 (52.9%) | 82 (31.8%) | 0.07 |
| G3 | 5 (10.4%) | 8 (24.2%) | 10 (19.6%) | 23 (17.4%) | 0.03 |
| Missing | 0 | 11 (33.3%) | 12 (23.5%) | 23 (17.4%) | <0.001 |
| LOCATION OF PRIMARY TUMOR |  |  |  |  |  |
| Cecum | 4 (8.4%) | 2 (6.1%) | 2 (4%) | 8 (6.1%) | 0.67 |
| Ascending Colon | 7 (14.6%) | 5 (15.2%) | 7 (13.7%) | 19 (14.4%) | 0.99 |
| Transverse Colon | 1 (2.1%) | 1 (3%) | 1 (2%) | 3 (2.3%) | 0.3 |
| Descending colon | 9 (18.8%) | 5 (15.3%) | 12 (23.5%) | 26 (19.7%) | 0.65 |
| Sigmoid Colon | 18 (37.5%) | 5 (15.3%) | 12 (23.5%) | 35(26.5%) | 0.07 |
| Rectum | 9 (18.8%) | 14 (42.4%) | 17 (33.3%) | 40 (30.3%) | 0.06 |
| Missing | 0 | 1 (3%) | 0 | 1 (0.8%) |  |
| NODAL STATUS OF PRIMARY |  |  |  |  |  |
| 0 | 12 (25%) | 11 (33.3%) | 12 (23.5%) | 35 (26.5%) | 0.72 |
| 1 | 22 (45.8%) | 11 (33.3%) | 26 (50.1%) | 59 (44.7%) | 0.18 |
| 2 | 12 (25%) | 11 (33.3%) | 10 (19.6%) | 33 (25%) | 0.46 |
| Missing | 2 (4.2%) | 0 | 3 (5.9%) | 5 (37.9%) | 0.44 |
| Metastatic CRC at diagnosis | 37 (77.1%) | 13 (39.4%) | 37 (72.5%) | 87 (65.9%) | 0.001 |
| Intrahepatic Progression | 32 (66.7%) | 15 (45.4%) | 32 (62.75%) | 79 (59.8%) | 0.16 |
| Extrahepatic Progression | 8 (16.7%) | 6 (18.2%) | 13 (25.5%) | 27 (20.5%) | 0.56 |
| Cancer-related Death | 9 (18.8%) | 6 (18.2%) | 6 (11.8%) | 21 (15.9%) | 0.6 |

| **Table S2 - Tumor Characteristics** | | | |  |  |
| --- | --- | --- | --- | --- | --- |
| **Variables** | **INSTITUTION A (N=78)** | **INSTITUTION B (N=40)** | **INSTITUTION C (N=95)** | **TOTAL (N=204)** | **P** |
| Size at ablation (mm)^†^ | 12.6±6.9 (4-30) | 21.6±7.8 (7-37) | 15±7.6 (3-35) | 15.4±8 (3-37) | <0.001 |
| Size at diagnosis (mm) | 12.8±7.6 (4-40) | 17.8±9.2 (7-42) | 14.9±8.4 (3-46) | 14.8±8.2 (3-46) | 0.02 |
| Largest lesion size at any time | 14.9 ±7.8 (5-40) | 24.3±10 (7-47) | 18.7±9.6 (3-46) | 18.4±9.5 (3-47) | <0.001 |
| Amount of delivered energy (W x s/mm)^†^ | 1779±700.5 | 1986.7±927.44 | 2653±1544.4 | 2199.4±545.5 | <0.001 |
| TIMING OF LESION APPEARANCE |  |  |  |  |  |
| Synchronous | 40 (51.3%) | 11 (27.5%) | 32 (33.7%) | 83 (39%) | 0.01 |
| Metachronous | 38 (48.7%) | 29 (72.5%) | 63 (66.3%) | 130 (61%) |  |
| Subcapsular Location | 16 (20.5%) | 12 (30%) | 26 (27.4%) | 54 (25.4%) | 0.45 |
| Proximity to Vessels >3mm | 20 (25.6%) | 25 (62.5%) | 34 (35.8%) | 79 (37.1%) | 0.01 |
| Time from last hepatic resection to ablation | 347,31±354,65 (92 – 1388) | 337±413.9 (5 – 1210) | 546,45±379,08 (168 – 1464) | 406.7±382.5 (5 – 1464) | 0.05 |
| Time from lesion discovery to ablation (days) | 150±144 (0-779) | 153.8±170 (0-742) | 172±165.6 (0-650) | 160.6±158.8 (0-779) | 0.5 |
| Chemotherapy after lesion discovery and before ablation | 48 (61.5%) | 20 (50%) | 44 (46.3%) | 112 (52.6%) | 0.1 |
| Time from last chemotherapy cycle to ablation (days) | 79,31±73,8 (9 – 381) | 64,4±54,12 (0 – 165) | 57,75±44,24 (14 – 141) | 68,17±60,55 (0 – 381) | 0.31 |
| ABLATION MARGIN |  |  |  |  |  |
| <5 mm | 35 (44.9%) | 12 (30%) | 20 (21%) | 67 (31.5%) | 0.004 |
| 5-10 mm | 27 (34.6%) | 20 (50%) | 26 (27.4%) | 73 (34.3%) | 0.04 |
| >10 mm | 14 (17.9%) | 7 (17.5%) | 43 (45.3%) | 64 (30%) | <0.001 |
| Incomplete ablation | 2 (2.6%) | 1 (2.5%) | 6 (6.3%) | 9 (4.2%) | 0.54 |
| Local Tumor Progression | 20 (25.6%) | 10 (25%) | 28 (29.5%) | 58 (27.2%) | 0.83 |
